# Publisher Correction: Trapping Phenomenon Attenuates the Consequences of Tipping Points for Limit Cycles

**DOI:** 10.1038/s41598-018-35962-8

**Published:** 2018-11-26

**Authors:** Everton S. Medeiros, Iberê L. Caldas, Murilo S. Baptista, Ulrike Feudel

**Affiliations:** 1 Institute of Physics, University of São Paulo, São Paulo, Brazil; 2 Institute for Complex Systems and Mathematical Biology, SUPA, University of Aberdeen, Aberdeen, United Kingdom; 3 Institute for Chemistry and Biology of the Marine Environment, Carl von Ossietzky University Oldenburg, Oldenburg, Germany

Correction to: *Scientific Reports* 10.1038/srep42351, published online 09 February 2017

This Article contains errors in Figure 2 where the labelling of the yellow and blue lines is incorrect and should read ‘S_1_’ and ‘S_2_’ respectively.

In addition,

“Amplitude (A)”

should read:

“Forcing Amplitude (A)”

Furthermore, a display error resulted in the blue S_2_ lines being truncated on the right-hand side of the Figure.

The correct Figure 2 appears below as Figure 1.

**Figure 1:**
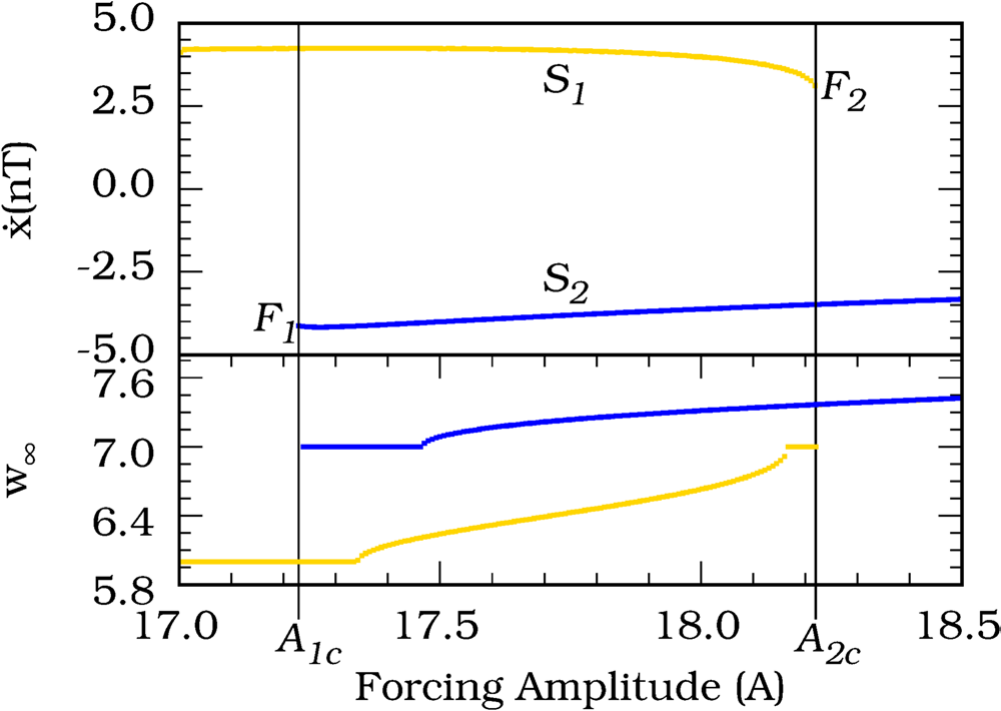
(Upper) Bifurcation diagram of the noise-free (σ = 0) Duffing oscillator showing a bistability of limit cycles. The different colors, blue and yellow, represent each limit cycle, *S*_2_ and *S*_1_, respectively. The state variable $\dot{x}$ (*nT*) is the *T*-shift map of the limit cycle variable, $\dot{x}$. The points *F*_1_ and *F*_2_ mark the parameters where catastrophic shift occurs, *A*_1*c*_ = 17.2295 and *A*_2*c*_ = 8.2250 are the corresponding critical parameter values. The other system parameters are settled in *d* = 0.3, *ω* = 0.5. (Bottom) The asymptotic generalized winding numbers, *w*_∞_, of each limit cycle in the parameter interval. The colors correspond to the respective limit cycles.

